# Supplementary material for: Affective neural signatures do not distinguish women with emotion dysregulation from healthy controls: A mega-analysis across three task-based fMRI studies
Source: Neuroimage Rep. 2021 May 29;1(2):100019. doi: 10.1016/j.ynirp.2021.100019 (PMC12172786; doi:10.1016/j.ynirp.2021.100019)
Supplement: Multimedia component 1 [file mmc1.docx]

**Emotion dysregulation is not explained by affective neural signatures:**

**A mega-analysis across three clinical studies**

**Supplemental Materials**

[**1. Supplemental Methods**](#_ca2zlm51lggu) 2

[1.1. Study 1](#_1tlumeqbl98n) 2

[1.1.1. Participants](#_iofp17m9cg84) 2

[1.1.2. Stimulus material and procedure](#_7bfdrm1wy1j7) 3

[1.1.3. Data acquisition and preprocessing](#_f2cvj8xwvne2) 3

[1.1.4. First-level analysis](#_nyyh9k7ask55) 4

[1.2. Study 2](#_vuw9m6ukrxzv) 4

[1.2.1. Participants](#_2dfazha43i2q) 4

[1.2.2. Stimulus material and procedure](#_6kcvocc3x4wn) 4

[1.2.3. Data acquisition and preprocessing](#_1316ql955tro) 5

[1.2.4. First-level analysis](#_ni5mg3yyune2) 6

[1.3. Study 3](#_1ga99agfyz31) 6

[1.3.1. Participants](#_5k7tn56wgcgt) 6

[1.3.2. Stimulus material and procedure](#_pre07rhk5tj0) 7

[1.3.3. Data acquisition and preprocessing](#_pp4lu5u3vatj) 7

[1.3.4. First-level analysis](#_q909pqvvedcn) 8

[1.4. Supplemental descriptive tables](#_2djx7k8ktnwq) 9

[1.4.2. Table S1](#_505r51smnq97) 9

[1.4.3. Table S2](#_p3odjydq1xip) 11

[1.4.4. Table S3](#_p3odjydq1xip) 13

[**2. Supplemental Results**](#_9pt614x8wwzq) 15

[2.1. Supplemental figures](#_s7eljgfqajyr) 15

[2.1.1. Figure S1](#_o6thg6i2zooj) 16

[2.1.2. Figure S2](#_6igqp7z2dahp) 17

[**3. References**](#_9pt614x8wwzq)  18

# 1. Supplemental Methods

## 1.1. Study 1

### 1.1.1. Participants

The current sample of 29 patients with BPD and 28 HC was recruited within a larger project on alterations in neural correlates of emotion regulation in BPD after DBT, which was registered as a clinical trial (German clinical trials register DRKS00000778). This project focused on longitudinal data, assessing on improvements after DBT versus treatment as usual, and several papers on alterations in structural and functional brain correlates were published before [(Niedtfeld et al., 2017; Schmitt et al., 2016; Winter et al., 2017)](https://paperpile.com/c/Zgwelv/DKpl+eAQU+4HBP), suggesting normalization of emotion regulation via reappraisal, and a reduced effect of painful stimulation as a dysfunctional attempt to regulate negative affect after psychotherapy. However, previous analyses excluded participants that did not take part at both scanning sessions, pre and post 12 weeks of psychotherapy. In the current analysis, we included all complete datasets that were acquired at the first assessment point.

Patients were recruited at specialized DBT inpatient treatment units at the Central Institute of Mental Health Mannheim and at Heidelberg University Hospital, at local outpatient treatment units, and via email contact to local psychotherapists. Patients received DBT treatment, individual psychotherapy, residential crisis intervention, pharmacotherapy, self-help groups, or no specialized care. For more information on demographic and clinical characteristics, see Supplemental Table S1. Study procedures were confirmed by the Ethics Board of the Medical Faculty Mannheim of the University of Heidelberg (Nr. 2010-243N-MA) and all subjects provided written informed consent before participation. All participants received monetary compensation of 12€/hour for their participation.

All BPD patients met DSM-IV diagnosis for BPD, including affective instability and NSSI during the last month prior to the first assessment. Diagnoses were assessed by trained clinical psychologists carrying out the German Versions of the Structured Clinical Interview for DSM-IV [(Wittchen et al., 1997)](https://paperpile.com/c/Zgwelv/iHXb), and the International Personality Disorder Examination [(Loranger et al., 1998)](https://paperpile.com/c/Zgwelv/LfP6). Symptom severity of BPD was assessed via the Zanarini Rating Scale for BPD [(Zanarini et al., 2003)](https://paperpile.com/c/Zgwelv/wRXU), and the Borderline Symptom List [(Bohus et al., 2009)](https://paperpile.com/c/Zgwelv/HUB1). Additionally, BPD patients were either unmedicated or had a constant medication. HC did not meet any lifetime psychiatric disorder and received no psychotropic medication. We further excluded participants with left-handedness, traumatic brain injury, lifetime schizophrenia or bipolar I disorder, mental or developmental disorders, substance dependence during the last year, drug consumption in the last two months, current severe depressive episode, and benzodiazepine use.

### 1.1.2. Stimulus material and procedure

The fMRI task was a well-validated emotion regulation task [(Ochsner et al., 2012)](https://paperpile.com/c/Zgwelv/LEVh) that were previously used in patients with BPD [(Krause-Utz et al., 2012; Niedtfeld et al., 2010; Schulze et al., 2011)](https://paperpile.com/c/Zgwelv/RDXL+HRhh+BCfH). The paradigm was designed to incorporate two within-subject factors (picture valence, regulation condition). Within three different runs, we incorporated different emotion regulation conditions: distract versus look, reappraise versus look, and painful temperature versus look. In each run, 72 experimental trials were presented, consisting of negative or neutral picture stimuli (each presented for 6s), which were selected from two standardized picture sets, the Emotional Picture Set [(Wessa et al., 2010)](https://paperpile.com/c/Zgwelv/08yI) and the International Affective Picture System [(Lang et al., 2005)](https://paperpile.com/c/Zgwelv/GvUg). We parallelized pictures with regard to valence and arousal between runs and conditions. For the current analysis, we extracted the look condition for each picture valence (i.e. 18 trials negative look, 18 trials neutral look, for each run), resulting in 108 trials for each participant. Between trials, participants saw a white fixation cross on a black screen, presented for a jittered time interval of 3 to 8 seconds. To monitor vigilance of the participants, 24 catch trials (i.e. the letter “O”) were included between experimental trials that required an immediate button press response.

### 1.1.3. Data acquisition and preprocessing

Brain images were acquired using a 3 Tesla MRI scanner (TRIO, Siemens Medical Systems, Erlangen, Germany) with a 32-channel head coil and a T2*- weighted gradient echo-planar imaging sequence (repetition time=2000ms, echo time=30ms, voxel size =3x3x3mm, matrix = 64x 64, number of slices = 36). A high-resolution T1-weighted structural scan was acquired for co-registration of functional images. Functional data were analyzed using SPM8 (Wellcome Department of Cognitive Neurology, London, United Kingdom). The echo-planar imaging time series were pre-processed according to custom practice. Procedures comprised slice time correction, spatial realignment, segmentation of T1 scan, coregistration onto T1 scan, normalization to the standard brain of the Montreal Neurological Institute space, resampling to 3 mm3 voxels, smoothing with a Gaussian kernel with a full-width at half maximum of 6 mm.

### 1.1.4. First-level analysis

On the individual level, we modeled four regressors of interest (using a canonical HRF), resembling the 2x2 factor levels (i.e. negative regulate, negative look, neutral regulate, neutral look), and seven regressors of no interest (button presses, six movement parameters). To correct for low-frequency fluctuations and global signal intensity variation, a high-pass filter of 128s was applied. The contrasts images for this study (i.e. negative look, neutral look) were entered into the second level analyses.

## 1.2. Study 2

### 1.2.1. Participants

We tested 22 healthy female participants and 20 participants with Borderline Personality Disorder (BPD). One additional participant was measured in the BPD group after a phone screening but had to be excluded afterwards because she did not fulfill at least 5 DSM-IV BPD criteria. One healthy participant had to be excluded because of excessive movements (translation >3 mm) and another subject could not be included in the analysis due to an incidental finding. This resulted in N = 20 healthy subjects in the fMRI analysis. Healthy participants reported no current and past DSM-IV Axis I syndrome or family history of neurological or psychiatric disorders, as confirmed by a structured clinical interview [(First et al., 1997)](https://www.zotero.org/google-docs/?mLfpZL). All participants were of Caucasian origin. Demographics and sample characteristics are provided in Table S2. Study procedures were confirmed by the Ethics Board of the Medical Faculty Mannheim of the University of Heidelberg (Nr. 2011-224N-MA) and all subjects provided written informed consent before participation. Compensation for expenses was 24 Euro.

### 1.2.2. Stimulus material and procedure

Participants underwent three fMRI-runs comprising the presentation of aversive pictures and scrambled versions of the same pictures as a baseline. All pictures were taken from standardized picture series [(Lang et al., 2008; Wessa et al., 2010)](https://www.zotero.org/google-docs/?upm0TM), where stimuli had been rated by representative samples using the Self-Assessment Manikin (SAM) affective rating system on a 9-point scale. Stimuli were chosen to have high ratings of arousal and low ratings of pleasure referring to a high level of negative valence, respectively, and depicted scenes such as accidents, suffering people, or war. Each picture was presented only once to each participant during the whole experiment. Each scanning run used a different mode of stimulus presentation, subsequently referred to as different experimental ‘designs’. Specifically, we employed an event related (ER) design (373 scans), an Single Picture Block (SPB) design (218 scans), and a multiple picture block (MPB) design (218 scans). In ER, 36 stimuli were presented for 2 s each with an adjacent inter-trial interval (ITI) of 20 ± 1 s to allow partial recovery of the BOLD signal. During the ITI, participants viewed a white fixation cross on a black background. SPB and MPB used a trial duration of 18 s and an ITI of 12 ± 3 s. In SPB, one stimulus was presented for the whole 18 s, whereas in MPB, three stimuli were presented consecutively for 6 s each, resulting in a set of 14 pictures in the former and 42 in the latter design. The ITI was jittered within the given range to ensure reduced predictability of picture onset and optimized sampling of the BOLD signal. Picture assignment to design-type as well as design-type order was counterbalanced and randomized between subjects, as was trial order with the restriction of ≤2 consecutive stimuli of the same valence. A difference in affective intensity between design-types was prevented by matching the mean normative valence and arousal ratings of stimuli in the different runs. Before the first run, subjects were instructed to look directly at the pictures during the entire duration of presentation and not to distract themselves by thinking about other things. Each run started with a written instruction (6 s) introducing the upcoming design-type in German (e.g., for SPB: ‘Now you will see 14 pictures for 18 s each’). After every run, subjects were asked to rate their current level of aversive tension. Stimuli were presented using Presentation software (Neurobehavioral Systems, Inc, Berkeley, USA) via a 40″ monitor located in the back of the scanner which was visible for subjects through a mirror placed on top of the head coil. After completion of the experiment, subjects were asked to rate the negative stimuli with regard to valence and arousal outside the MRI suite.

### 1.2.3. Data acquisition and preprocessing

FMRI data were acquired on a 3 TeslaMRI Scanner (Magnetom Trio with TIM technology, Siemens Medical Service, Erlangen, Germany) equipped with a 32 channel head coil. Functional images of the BOLD contrast were acquired with gradient echo T2* weighted echo-planar imaging sequence (TE = 30 ms, TR = 2 s, FOV = 220 mm × 220 mm, matrix size=64 × 64, flip angle=80°). A volume comprised 36 slices in AC–PC orientation with a thickness of 3 mm and slice gap of 1 mm. Participants' heads were lightly restrained using soft pads to prevent head movement. A T1-weighted anatomical image was also recorded (TE = 3.03 ms, TR = 2.3 s, 192 slices and FOV = 256 mm × 256 mm, matrix size 256 × 256, slice thickness = 1 mm). FMRI data were analyzed with SPM8 (Wellcome Department of Cognitive Neurology, London, UK). Before preprocessing of functional data, 5 initial volumes were discarded to avoid T1 effects. A slice timing correction of the functional scans was performed with reference to the 18th slice to correct for differences in acquisition time between slices. Realignment of functional images to the mean functional image was performed using a rigid body transformation. The required transformation matrix for the alignment of functional and anatomical (T1) images was estimated via the SPM12 coregister module. The T1 image was segmented into six tissue types using the ICBM template (Montreal Neurological Institute (MNI) system) for the normalization. The received normalization parameters were used to transform the functional images into MNI space. Normalized functional images were finally smoothed with a kernel of 8 mm (FWHM).

### 1.2.4. First-level analysis

On the single-subject-level, we performed a separate General Linear Model (GLM) analysis for each run, resulting in one model for each design, i.e., ER, SPB, and MPB. In each analysis, 3 conditions were modeled: ‘negative’ (negative condition) as well as ‘scrambled’ (neutral condition) picture presentation, and the instruction (duration=6 s) at the beginning of the trial. The ITI period served as an implicit baseline. A high-pass filter (128 s) was added to the GLM to remove slow signal drifts and serial correlations were accounted for using an auto-regressive (AR(1)) model. All regressors were convolved with the HRF implemented in SPM12.

## 1.3. Study 3

### 1.3.1. Participants

The sample comprised 62 women with cPTSD and 33 healthy controls. cPTSD participants were recruited from a larger randomized controlled trial (German Clinical Trials Register: [DRKS00005578](https://www.drks.de/drks_web/navigate.do?navigationId=trial.HTML&TRIAL_ID=DRKS00005578)) on the efficacy of dialectical behavior therapy and cognitive processing therapy in cPTSD after childhood abuse [(Bohus et al., 2020)](https://paperpile.com/c/Zgwelv/gzLy). They underwent fMRI measurements between randomization and the first therapy session. All cPTSD patients met DSM-5 diagnosis for PTSD and met at least three criteria for borderline personality disorder, including criterion six for emotional instability. Diagnoses were assessed by trained clinical psychologists carrying out the German Versions of the Structured Clinical Interview for DSM-IV [(Wittchen et al., 1997)](https://paperpile.com/c/Zgwelv/iHXb) and the international personality disorder examination [(Loranger et al., 1998)](https://paperpile.com/c/Zgwelv/LfP6).

Healthy control participants were recruited with advertisements in local newspapers, flyers and over the internet. The study was approved by the Ethical Board II of Heidelberg University (Nr.: 2013-635N-MA), Germany, and was conducted according to the Declaration of Helsinki at the Central Institute of Mental Health in Mannheim, Germany. Participants provided written consent after the procedures had been fully explained. All participants received monetary compensation of 12€/h for their participation.

### 1.3.2. Stimulus material and procedure

Participants were shown negative versus neutral pictures from the International Affective Picture System (IAPS; [(Lang et al., 2005)](https://paperpile.com/c/Zgwelv/GvUg), which were presented within a Sternberg working memory task. Participants saw sixteen pictures which were preselected as negative or neutral based on arousal and valence ratings in the general population. Pictures in the negative condition included negatively arousing interpersonal scenes on physical and sexual violence, emotional neglect, or mutilation. Neutral pictures were matched to negative pictures regarding the number of persons and complexity of the scene to control for potentially confounding differences in visual information processing. In the present study, only the contrast between negative and neutral pictures was used.

The task consisted of 48 trials, each starting with the presentation of three uppercase letters (memoranda, 1000 ms). After a delay interval (1500 ms), again three letters (probe, 2000 ms) were presented, which participants had to compare with the memoranda. Participants had to press a “yes” button whenever they recognized a target, i.e. a letter previously presented in the memorandum. In half of the trials, a target (one of the three memoranda) was present in the probe. During the delay interval, either a fixation cross or a picture stimulus (negative or neutral) was presented. The resting phase between the trials was jittered to prevent temporal correlation. We used the Software Presentation (Neurobehavioral Systems) to present stimuli and record behavioral data.

### 1.3.3. Data acquisition and preprocessing

Brain images were acquired using a 3 Tesla MRI scanner (TRIO, Siemens Medical Solutions, Erlangen, Germany) with a 32-channel head coil. Using three-dimensional magnetization-prepared rapid-acquisition gradient echo (MPRAGE; T1-weighted contrast, voxel size 111 mm³), a high-resolution anatomical scan was acquired for each participant as an individual template for the functional data. T2-weighted gradient echo planar imaging was used for measurement of the blood oxygen level-dependent (BOLD) signal [EPI, T2-weighted contrast, field of view = 192192 mm, voxel size 333 mm³, 6464 voxel matrix, flip angle 80°, echo time (TE) = 30 ms, repetition time (TR) = 2000 ms], with 36 transversal slices (3 mm, descending) covering the entire brain. The ﬁrst four scans were discarded to minimize T1 effects. Head movement artefacts and scanning noise were restricted using head cushions and headphones.

Functional imaging data were processed using standard procedures implemented in Statistical Parametric Mapping (SPM 12; Welcome Department of Cognitive Neurology, London, UK; www.fil.ion.ucl.ac.uk/spm/). The EPI time series were pre-processed according to custom practice, including slice time correction, spatial realignment, segmentation of T1 scan, co-registration onto T1 scan, and normalization to the standard brain of the Montreal Neurological Institute (MNI) space. We did not have to exclude subjects due to excessive head motion (exclusion criterion for head motion was 3mm in each direction).

### 1.3.4. First-level analysis

Whole-brain voxel-wise regression weights of the regressors for negative and neutral pictures were calculated using the first-level analysis procedure from SPM 12. We modelled the neural response with three regressors of interest (negative pictures, neutral pictures, fixation cross) and six motion regressors. The regressors were convolved with the canonical hemodynamic response function. A contrast image between negative and neutral pictures was calculated by subtracting the beta image of the negative picture regressor from the beta image of the neutral picture regressor.

## 1.4. Supplemental descriptive tables

### 1.4.2. Table S1

| Table S1 | |  |  |
| --- | --- | --- | --- |
| *Sample descriptives for study 1* | |  |  |
|  |  | BPD | HC |
|  |  | N = 28 | N = 29 |
| Demographics | |  |  |
|  | Age mean (SD) | 25.89 (6.82) | 26.83 (8.21) |
|  | Memory span mean (SD) | 15.11 (3.14) | 16.62 (3.74) |
| School education N (%) | |  |  |
|  | School-leaving qualification | 5 (17.9) | 1 (3.4) |
|  | Secondary school-leaving qualification | 9 (32.1) | 15 (51.7) |
|  | General matriculation standard | 12 (42.9) | 13 (44.8) |
|  | Other | 2 (7.1) | 0 |
| Profession N (%) | |  |  |
|  | None | 12 (42.9) | 11 (37.9) |
|  | Vocational Training | 13 (46.4) | 13 (44.8) |
|  | University/College | 2 (7.1) | 3 (10.3) |
|  | Missing | 1 (3.6) | 2 (6.9) |
| Clinical Characteristics M (SD) | |  |  |
|  | ZAN-BPD | 15.64 (6.58) | 0.48 (1.27) |
|  | DERS | 125.59 (24.56) | 61.10 (14.19) |
|  | BDI | 27.67 (11.16) | 1.90 (3.32) |
|  | STAI-State | 56.58 (11.43) | 29.75 (6.92) |
|  | STAI-Trait | 60.35 (9.15) | 29.69 (8.00) |
|  | BSL | 1.89 (0.80) | 0.21 (0.16) |
|  | RSQ-D | 18.06 (7.24) | 5.32 (3.15) |
|  | FDS | 23.91 (15.22) | 2.22 (2.21) |
| Current Comorbidities N (%) | |  |  |
|  | Posttraumatic Stress Disorder | 12 (42.1) |  |
|  | Major Depressive Disorder | 8 (28.6) |  |
|  | Anxiety Disorder | 6 (21.4) |  |
|  | Obsessive-Compulsive Disorder | 4 (14.3) |  |
|  | Bipolar II | 1 (3.6) |  |
|  | Somatoform Disorder | 1 (3.6) |  |
|  | Eating Disorder | 13 (46.4) |  |
|  | Substance Dependency | 5 (17.9) |  |
|  | Substance Abuse | 4 (14.3) |  |
|  | Other Current DSM-V Disorders | 2 (7.1) |  |
| Psychotropic Medication N (%) | |  |  |
|  | Total | 4 (14.3) |  |
|  | SSRI | 4 (14.3) |  |
|  | Atypical Neuroleptics | 2 (7.1) |  |
|  | Other | 1 (3.6) |  |
|  | Unmedicated | 24 (85.71) |  |

*Note.* ZAN-BPD = Zanarini Rating Scale for BPD, DERS = Difficulties in Emotion Regulation Scale, BDI = Beck Depression Inventory, STAI = State-Trait-Anxiety Inventory, BSL = Borderline Symptom List, RSQ-D = Response Style Questionnaire - German Version, FDS = Questionnaire for Dissociative Symptoms (German).

###

### 1.4.3. Table S2

Table S2

*Sample descriptives for study 2*

|  |  | BPD | HC |
| --- | --- | --- | --- |
|  |  | *N* = 20 | *N* = 20 |
| *Demographics* | |  |  |
|  | Age mean (*SD*) | 27.6 (6.43) | 26.1 (4.53) |
| *School Education N (%)* | |  |  |
|  | School-leaving qualification | 0 (0) | 0 (0) |
|  | Secondary school-leaving qualification | 7 (35) | 2 (10) |
|  | General matriculation standard | 13 (65) | 18 (90) |
| Picture Ratings M (SD) | |  |  |
| Valence | | 3.68 (0.31) | 3.77 (0.50) |
| Arousal | | 2.91 (0.67) | 3.03 (0.61) |
| Clinical Characteristics M (SD) | |  |  |
| *DERS* | | 123.11 (15.04) | 64.4 (17.32) |
| *BDI* | | 27.5 (9.29) | 3 (3.61) |
| *STAI-State* | | 55.78 (7.46) | 30.5 (7.80) |
| *STAI-Trait* | | 60.5 (7.76) | 32.65 (9.50) |
| *BSL* | | 2.19 (0.72) | 0.1 (0.12) |
| *FDS* | | 24.39 (15.39) | 3.00 (2.01) |
| *CTQ* | | 61.06 (18.77) | 32.7 (9.71) |
| *Picture Rating: Valence* | | 3.68 (0.31) | 3.77 (0.50) |
| *Picture Rating: Arousal* | | 2.91 (0.67) | 3.03 (0.61) |
| *ZAN-BPD* | | 11.75 (4.87) | 0.89 (1.91) |
| *Current Comorbidities N (%)* | |  |  |
|  | Posttraumatic Stress Disorder | 5 (25) |  |
|  | Depressive Disorder | 13 (65) |  |
|  | Anxiety Disorder | 5 (25) |  |
|  | Panic Disorder | 2 (10) |  |
|  | Obsessive-Compulsive Disorder | 1 (5) |  |
|  | Somatoform Disorder | 3 (15) |  |
|  | Eating Disorder | 3 (15) |  |
|  | Substance Abuse | 2 (10) |  |
| *Psychotropic Medication N* (%) | |  |  |
|  | SSRI | 2 (10) |  |
|  | Unmedicated | 18 (90) |  |

*Note.* DERS = Difficulties in Emotion Regulation Scale, BDI = Beck Depression Inventory, STAI = State-Trait Anxiety Inventory, BSL = Borderline Symptom List, FDS = Dissociative Experiences Scale, CTQ = Childhood Trauma Questionnaire, ZAN-BPD = Zanarini Rating Scale for BDP. Picture ratings were only provided for images in the negative condition and not for scrambled images in the control condition.

### 1.4.4. Table S3

Table S3

*Sample descriptives for study 3*

|  |  | cPTSD | HC |
| --- | --- | --- | --- |
|  |  | *N* = 62 | *N* = 33 |
| *Demographics M (SD)* | |  |  |
|  | Age | 35.15 (11.40) | 32.30 (8.42) |
| School education in years | | 10.52 (1.40) | 11.33 (1.05) |
| *Picture Ratings M (SD)* | |  |  |
| Valence Negative | | 4.47 (0.34) | 4.38 (0.51) |
| Valence Neutral | | 2.70 (0.46) | 2.57 (0.54) |
| Arousal Negative | | 3.80 (0.85) | 3.31 (0.78) |
| Arousal Neutral | | 1.58 (0.48) | 1.15 (0.20) |
| *Clinical Characteristics M (SD)* | |  |  |
| *DTS* | | 80.26 (18.16) |  |
| *ZAN-BPD* | | 10.90 (5.40) |  |
| *DERS* | | 126.26 (21.11) | 60.12 (16.50) |
| *BDI* | | 34.42 (9.77) | 4.32 (4.94) |
| *STAI-State* | | 58.10 (10.30) | 29.85 (5.86) |
| *STAI-Trait* | | 62.32 (7.09) | 29.79 (7.72) |
| *BSI GSI* | | 1.86 (0.65) | 0.25 (0.24) |
| *CTQ* | | 14.86 (3.68) | 6.09 (5.33) |
| *GAF* | | 50.58 (7.90) | 91.13 (8.27) |
| *FDS* | | 22.56 (12.39) | 2.65 (2.40) |
| *Current Comorbidities N (%)* | |  |  |
|  | Posttraumatic Stress Disorder | 62 (100) |  |
|  | Affective Disorders | 40 (64.5) |  |
|  | Anxiety Disorders | 38 (61.3) |  |
|  | Obsessive-Compulsive Disorder | 6 (9.7) |  |
|  | Bipolar II | 0 |  |
|  | Somatoform Disorder | 5 (8.1) |  |
|  | Eating Disorder | 17 (27.4) |  |
|  | Substance Dependency | 0 |  |
|  | Substance Abuse | 0 |  |
|  | Borderline Personality Disorder | 33 (53.2) |  |
| *Psychotropic Medication N* (%) | |  |  |
|  | Total | 26 (56.7) |  |
|  | SSRI | 15 (25.0) |  |
|  | SNRI | 13 (21.7) |  |
|  | Other Antidepressants | 10 (16.7) |  |
|  | Atypical Neuroleptics | 12 (20.0) |  |
|  | Other Psychotropic Medication | 3 (5.0) |  |
|  | Unmedicated | 34 (43.3) |  |

*Note.* DTS = Davidson Trauma Scale, ZAN-BPD = Zanarini Rating Scale for BDP, DERS = Difficulties in Emotion Regulation Scale, BDI = Beck Depression Inventory, STAI = State-Trait Anxiety Inventory, BSI GSI = Brief Symptom Inventory Global Severity Index, GAF = Global Assessment of Functioning, CTQ = Child Trauma Questionnaire, FDS = Dissociative Experiences Scale. Picture ratings were provided for pictures in both the negative and the neutral condition.

## 1.5 Supplemental Figures

### 1.4.1 Figure S1


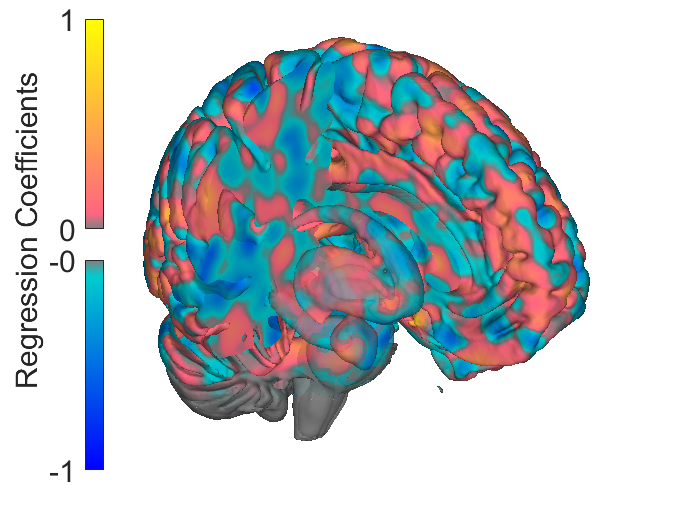


Figure S1. Exemplary illustration of the beta-weight map underlying the PINES signature. Coloring represents sign and magnitude of regression weights for each voxel. For example, high positive regression weights are assigned to bright-yellow voxels, indicating that larger activity in this voxel contributes to higher self-ratings of negative affect.

# 2. Supplemental Results

## 2.1. Supplemental figures

### 2.1.1. Figure S2


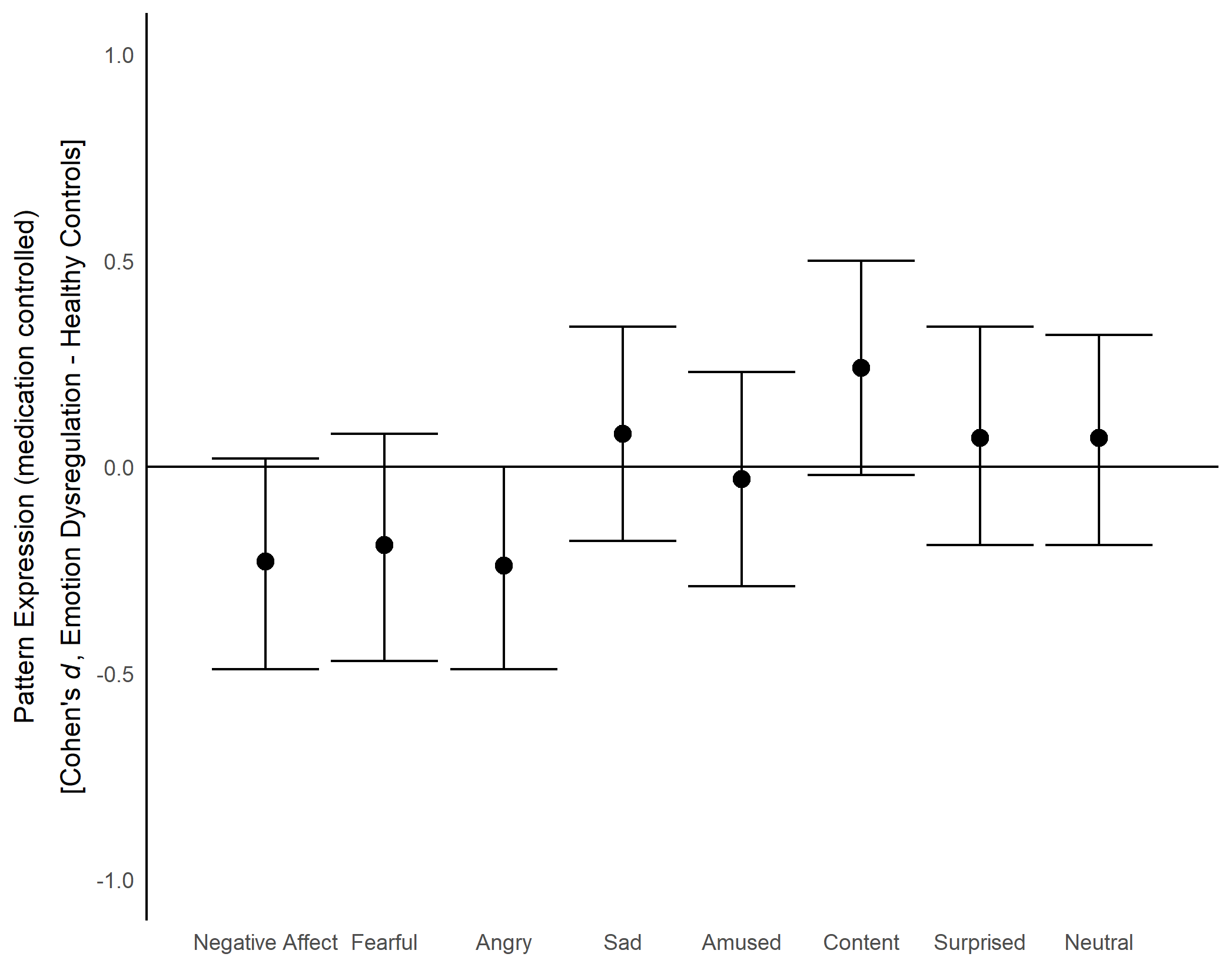


Figure S2. Mega-analytic group effects on pattern expression for the contrast negative versus neutral, controlling for medication status (medicated vs unmedicated). Error bars show 95% confidence intervals.

### 2.1.2. Figure S3


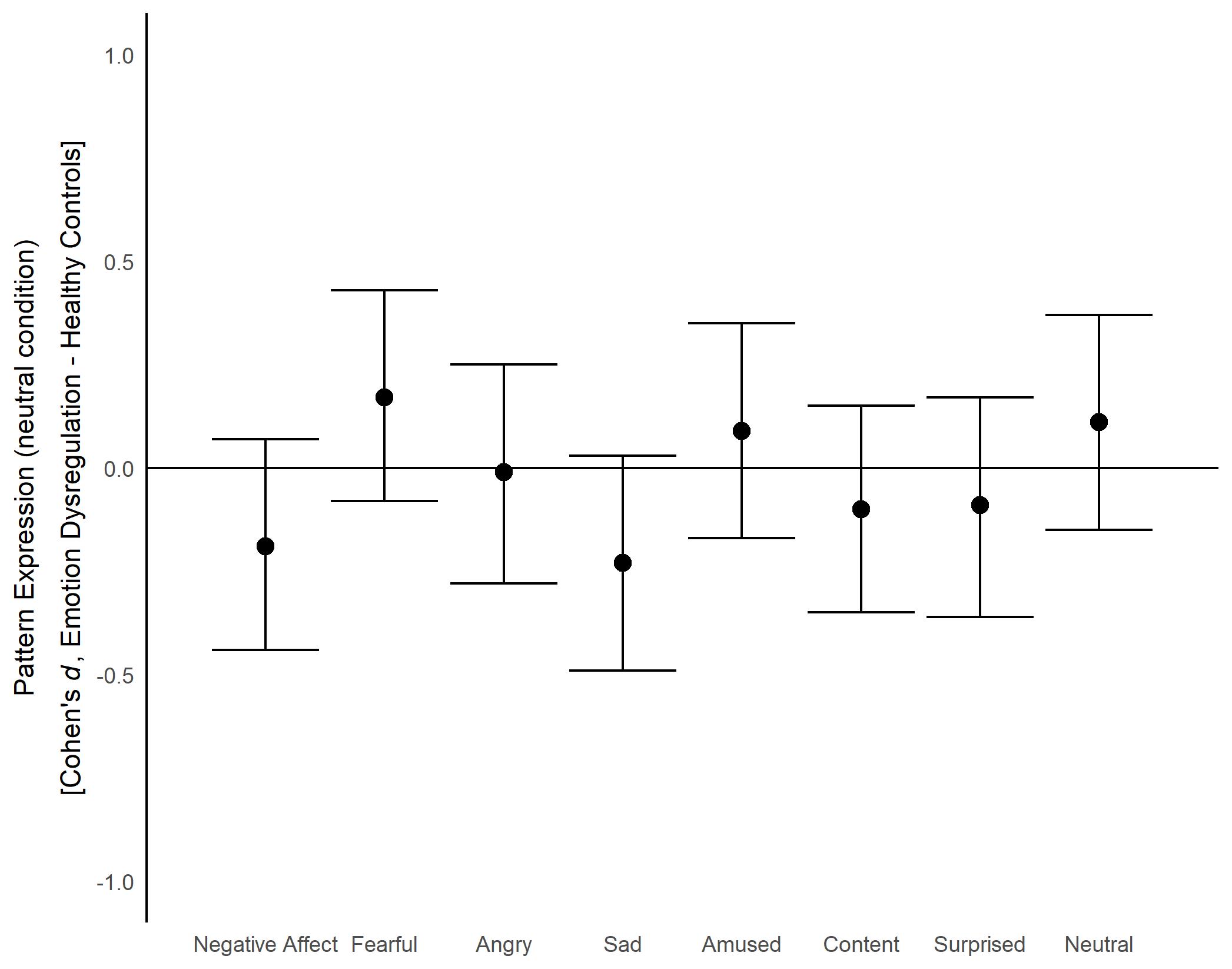


Figure S3. Mega-analytic group effects on pattern expression in the neutral baseline condition. Error bars show 95% confidence intervals.

### Figure S4


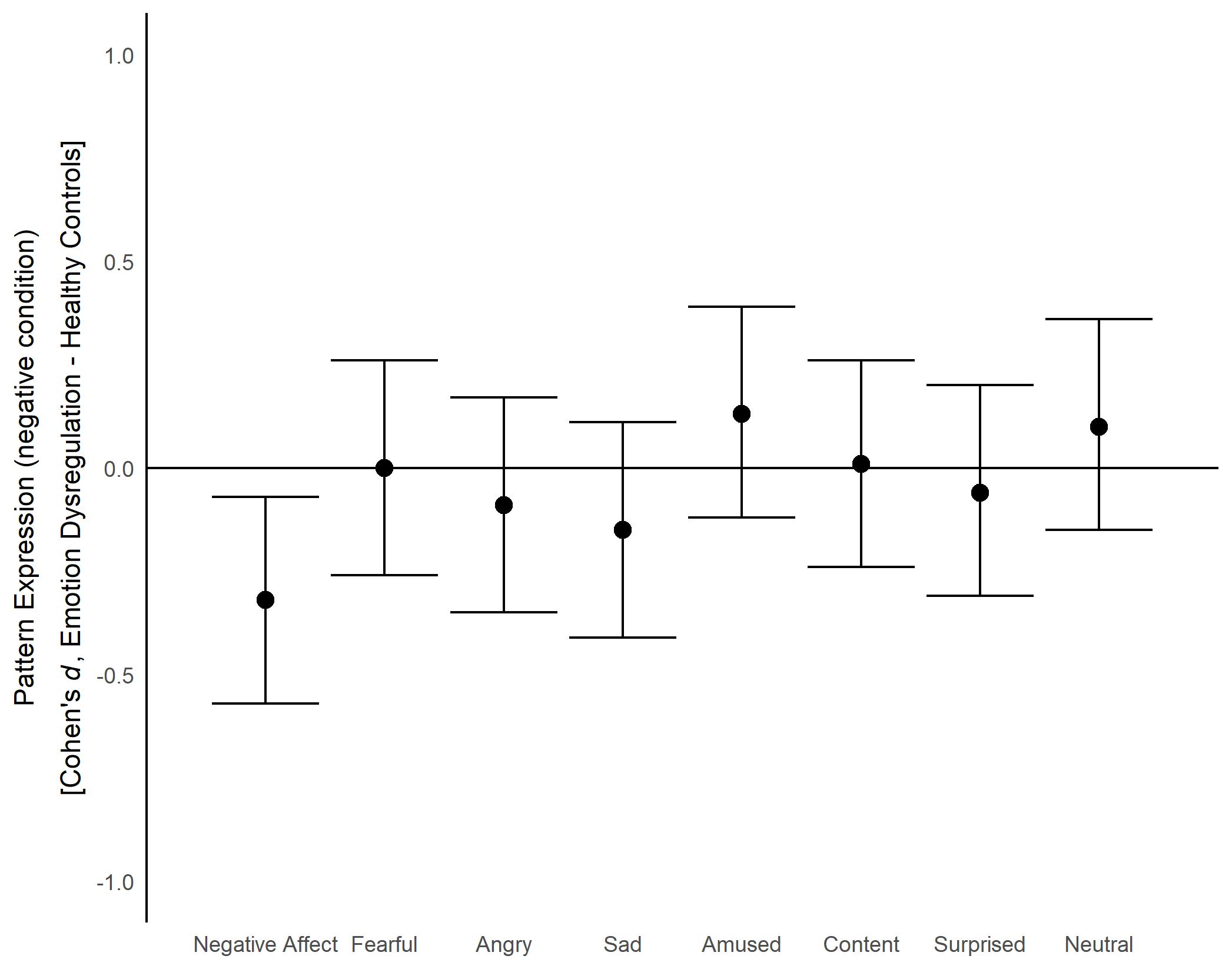


Figure S4. Mega-analytic group effects on pattern expression in the neutral baseline condition. Error bars show 95% confidence intervals.

# 3. References

[Bohus, M., Kleindienst, N., Hahn, C., Müller-Engelmann, M., Ludäscher, P., Steil, R., Fydrich, T., Kuehner, C., Resick, P. A., Stiglmayr, C., Schmahl, C., & Priebe, K. (2020). Dialectical Behavior Therapy for Posttraumatic Stress Disorder (DBT-PTSD) Compared With Cognitive Processing Therapy (CPT) in Complex Presentations of PTSD in Women Survivors of Childhood Abuse. In *JAMA Psychiatry* (Vol. 77, Issue 12, p. 1235). https://doi.org/](http://paperpile.com/b/Zgwelv/gzLy)[10.1001/jamapsychiatry.2020.2148](http://dx.doi.org/10.1001/jamapsychiatry.2020.2148)

[Bohus, M., Kleindienst, N., Limberger, M. F., Stieglitz, R.-D., Domsalla, M., Chapman, A. L., Steil, R., Philipsen, A., & Wolf, M. (2009). The short version of the Borderline Symptom List (BSL-23): development and initial data on psychometric properties. *Psychopathology*, *42*(1), 32–39.](http://paperpile.com/b/Zgwelv/HUB1)

[Krause-Utz, A., Oei, N. Y. L., Niedtfeld, I., Bohus, M., Spinhoven, P., Schmahl, C., & Elzinga, B. M. (2012). Influence of emotional distraction on working memory performance in borderline personality disorder. *Psychological Medicine*, *42*(10), 2181–2192.](http://paperpile.com/b/Zgwelv/RDXL)

[Lang, P. J., Bradley, M. M., & Cuthbert, B. N. (2005). *International Affective Picture System (IAPS): Digitized Photographs, Instruction Manual and Affective Ratings. (Technical Report a-6 Ed.)*. University of Florida.](http://paperpile.com/b/Zgwelv/GvUg)

[Loranger, A. W., Sartorius, N., Andreoli, A., Berger, P., Buchheim, P., Channabasavanna, S. M., Coid, B., Dahl, A. A., Diekstra, R. F. W., Ferguson, B., & Others. (1998). Deutschsprachige Fassung der International Personality Disorder Examination: IPDE.](http://paperpile.com/b/Zgwelv/LfP6) *[Genf, WHO](http://paperpile.com/b/Zgwelv/LfP6)*[.](http://paperpile.com/b/Zgwelv/LfP6)

[Niedtfeld, I., Schmitt, R., Winter, D., Bohus, M., Schmahl, C., & Herpertz, S. C. (2017). Pain-mediated affect regulation is reduced after dialectical behavior therapy in borderline personality disorder: a longitudinal fMRI study. *Social Cognitive and Affective Neuroscience*, *12*(5), 739–747.](http://paperpile.com/b/Zgwelv/DKpl)

[Niedtfeld, I., Schulze, L., Kirsch, P., Herpertz, S. C., Bohus, M., & Schmahl, C. (2010). Affect regulation and pain in borderline personality disorder: a possible link to the understanding of self-injury. *Biological Psychiatry*, *68*(4), 383–391.](http://paperpile.com/b/Zgwelv/HRhh)

[Ochsner, K. N., Silvers, J. A., & Buhle, J. T. (2012). Functional imaging studies of emotion regulation: a synthetic review and evolving model of the cognitive control of emotion. *Annals of the New York Academy of Sciences*, *1251*, E1–E24.](http://paperpile.com/b/Zgwelv/LEVh)

[Schmitt, R., Winter, D., Niedtfeld, I., Herpertz, S. C., & Schmahl, C. (2016). Effects of Psychotherapy on Neuronal Correlates of Reappraisal in Female Patients With Borderline Personality Disorder. *Biological Psychiatry. Cognitive Neuroscience and Neuroimaging*, *1*(6), 548–557.](http://paperpile.com/b/Zgwelv/eAQU)

[Schulze, L., Domes, G., Krüger, A., Berger, C., Fleischer, M., Prehn, K., Schmahl, C., Grossmann, A., Hauenstein, K., & Herpertz, S. C. (2011). Neuronal correlates of cognitive reappraisal in borderline patients with affective instability. *Biological Psychiatry*, *69*(6), 564–573.](http://paperpile.com/b/Zgwelv/BCfH)

[Wessa, M., Kanske, P., Neumeister, P., Bode, K., Heissler, J., & Schönfelder, S. (2010). EmoPics: Subjektive und psychophysiologische Evaluation neuen Bildmaterials für die klinisch-bio-psychologische Forschung.](http://paperpile.com/b/Zgwelv/08yI) *[Zeitschrift Für Klinische Psychologie Und Psychotherapie](http://paperpile.com/b/Zgwelv/08yI)*[, *39*(Suppl. 1/11), 77.](http://paperpile.com/b/Zgwelv/08yI)

[Winter, D., Niedtfeld, I., Schmitt, R., Bohus, M., Schmahl, C., & Herpertz, S. C. (2017). Neural correlates of distraction in borderline personality disorder before and after dialectical behavior therapy. *European Archives of Psychiatry and Clinical Neuroscience*, *267*(1), 51–62.](http://paperpile.com/b/Zgwelv/4HBP)

[Wittchen, H. U., Wunderlich, U., Gruschwitz, S., & Zaudig, M. (1997). SKID-I Strukturiertes Klinisches Interview für DSM-IV, Achse I.](http://paperpile.com/b/Zgwelv/iHXb) *[Göttingen: Hogrefe](http://paperpile.com/b/Zgwelv/iHXb)*[.](http://paperpile.com/b/Zgwelv/iHXb)

[Zanarini, M. C., Vujanovic, A. A., Parachini, E. A., Boulanger, J. L., Frankenburg, F. R., & Hennen, J. (2003). Zanarini Rating Scale for Borderline Personality Disorder (ZAN-BPD): a continuous measure of DSM-IV borderline psychopathology. *Journal of Personality Disorders*, *17*(3), 233–242.](http://paperpile.com/b/Zgwelv/wRXU)

[First, M.B., Spitzer, R.L., Gibbon, M., Williams, J.B.W., 1997. User’s Guide for the Structured Clinical Interview for Dsm-IV Axis I Disorders: Scid-1 Clinician Version, 1 edition. ed. American Psychiatric Publishing, Arlington.](https://www.zotero.org/google-docs/?UnR7w0)
